# Portable Electrochemical DNA Sensors Based on Gene Amplification Reactions to Screen and Identify Pathogen and SNPs

**DOI:** 10.3390/s22051865

**Published:** 2022-02-26

**Authors:** Eiichi Tamiya

**Affiliations:** 1 Advanced Photonics and Biosensing Open Innovation Laboratory, National Institute of Advanced Industrial Science and Technology, 2-1 Yamadaoka, Suita, Osaka 565-0871, Japan; tamiya@ap.eng.osaka-u.ac.jp; 2 Institute of Scientific and Industrial Research, Osaka University, 8-1 Mihogaoka, Ibaraki, Osaka 567-0047, Japan

**Keywords:** genetic sensor, electrochemical sensor, printed electrode, POCT (point-of-care testing) diagnosis, PCR, LAMP, influenza virus, SNPs, periodontal disease

## Abstract

In this paper, we introduce portable sensors based on genetic measurements that can be used in the field for the diagnosis of infectious diseases and disease risk based on SNPs (single nucleotide polymorphisms). In particular, the sensors are based on electrochemical measurements that can be performed with printed electrodes and small measuring devices. Indicator molecules that can bind to nucleic acid molecules in various ways are already known, and some of these molecules have electrochemical activity. First, we investigated the change in their electrochemical responses in a solution system. As a result, we searched for nucleic acid-binding molecules whose current value changes in the presence of DNA. In addition, when we measured the change in the current value, associated with the amplification of specific genes, such as PCR (polymerase chain reaction) and LAMP (loop-mediated isothermal amplification), we found that the current value decreased with the number of amplifications, indicating that specific genes can be monitored electrochemically. Based on this principle, we showed that pathogenic microorganisms and viruses, such as Salmonella, O157 *E. coli*, hepatitis B virus, periodontal disease bacteria, antibiotic-resistant bacteria and influenza virus, were able to be measured. The method was also applied to the diagnosis of SNPs, such as ApoE (apolipoprotein E), which is a risk factor for Alzheimer’s disease. Rapid PCR was available with a microfluidic device, and a simple method was also presented with the isothermal amplification of LAMP.

## 1. Advantages of Electrochemical Biosensors

**Figure 1:**
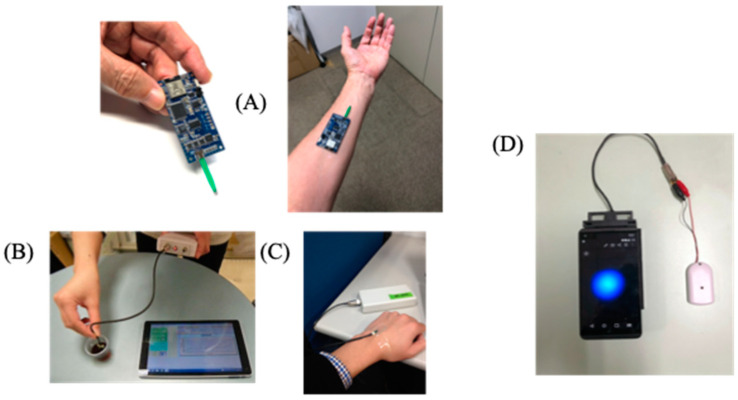
Electrochemical devices with compact, lightweight, wearable wireless capability. (**A**) One board electrochemical (EC) device with wireless communication by Bluetooth; (**B**) On-site electrochemical monitoring with the portable EC device; (**C**) Wearable monitoring sweat lactate on the skin; (**D**) Smartphone based electrochemiluminescence measurement.

Electrochemical biosensors are mainly known by potential and amperometric methods, which use the basic potential and current as indicators. In both methods, the biomolecules to be measured are specifically recognized by the molecules, and the resulting potential or current changes are sensed. For the potential method, biosensors that use ion electrodes and graphene FETs have been developed. Although highly sensitive measurements are possible, they are susceptible to non-specific molecules when measuring biological samples, such as blood and saliva, and there are large hurdles to their practical use as POCT biosensors, such as in direct measurements, without pretreatment. The amperometric method has the advantage that it can directly measure redox molecules derived from recognition reactions, followed by the transfer of electrons to/from electrodes. This is particularly evident in blood glucose sensors, which were the first biosensors to be implemented for practical use. Many biosensors that use a third phenomenon as an indicator, in addition to the potential and current induced by electrochemical methods, have also been developed, such as EIS (electrochemical impedance), electrochemiluminescence, electrochemical SERS (surface-enhanced Raman spectroscopy) and electrochemical SPR (surface plasmon resonance). As can be observed from the history of biosensors, electrochemistry was the beginning of biosensor research, as shown by the title of The Enzyme Electrode published in Nature by Updike and Hicks [1]. Currently, the following advantages of electrochemical biosensors have been recognized: (a) there have been many reports on electrochemical systems for various biosensors, such as enzyme sensors, immunosensors, gene sensors and cell sensors; (b) they are not affected by the background, such as coloration and fluorescence, in the sample; (c) miniaturization of the measurement device (e.g., wearable) is easy; (d) mass production of electrodes is easy (e.g., printing electrodes) and cost effective; (e) electrochemiluminescence can be used as an ultra-sensitive method. Electrochemical biosensors are also considered to be extremely advantageous in enabling personal use, and linking to POCT (point-of-care testing) and IoT for digital health. Carbon and gold electrodes are often used as working and counter electrodes in biosensors, while Ag/AgCl electrodes are often used as reference electrodes. In order to screen print these electrode materials, they are mixed with a polymeric material, which has similar viscosity to that of ink. Therefore, a conductive processing treatment is required for the final product. Ceramic, plastic and paper materials can also be used as printing substrates, and transparent and flexible printed electrodes can be produced. These printed electrodes can be mass produced at low cost (Supplementary Materials Figure S1), and are also useful for safety-critical applications, such as the measurement of biological samples. The electrochemical measuring board can be connected to the control unit and the data processing unit via wireless communication with a notebook PC. It is also possible to measure the concentration of lactic acid in sweat by attaching an electrochemical measuring board. More recently, small devices have been developed that can measure electrochemiluminescence, and that combine electrochemistry with a smart built-in smartphone camera (Figure 1).

## 2. Portable Electrochemical Gene Sensors

**Figure 2:**
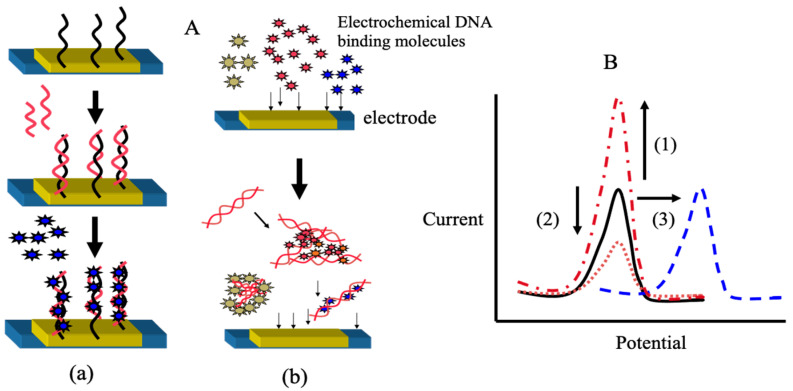
Electrochemical gene sensors with coupling with electrochemical DNA binding molecules (**A**): Scheme with (**a**) and without (**b**) immobilized DNA probes onto electrodes; (**B**): Type of electrochemical responses based on changes of current and potential. (1) Current increases with the amount of hybrid formed; (2) Current decreases with the amount of DNA; (3) Potential change with the amount of DNA.

**Figure 3:**
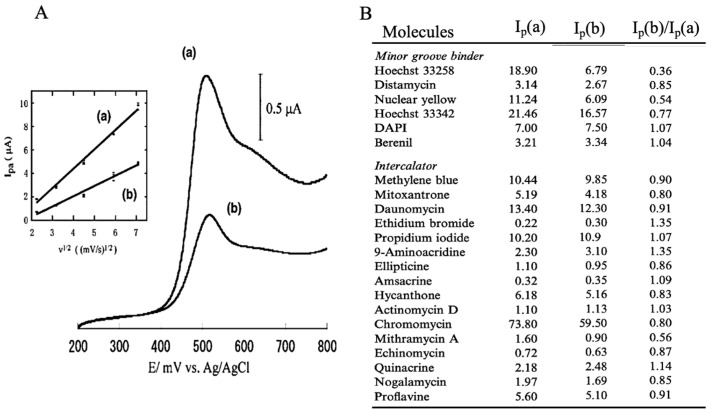
Comparison of electrochemical responses between DNA and various DNA-binding electrochemically active molecules [16] (**A**): Cyclic voltammograms with electrochemical indicator alone (a) and in the presence of DNA (b). (**B**): Comparison of I_p_(a), I_p_(b) and I_p_(b)/I_p_(a) among DNA-binding molecules. I_p_(a) and I_p_(b) indicated peak currents at (a) and (b) respectively.

**Figure 4:**
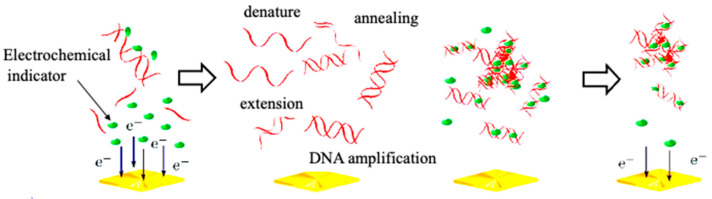
Scheme of electrochemical change linked with PCR amplification.

**Figure 5:**
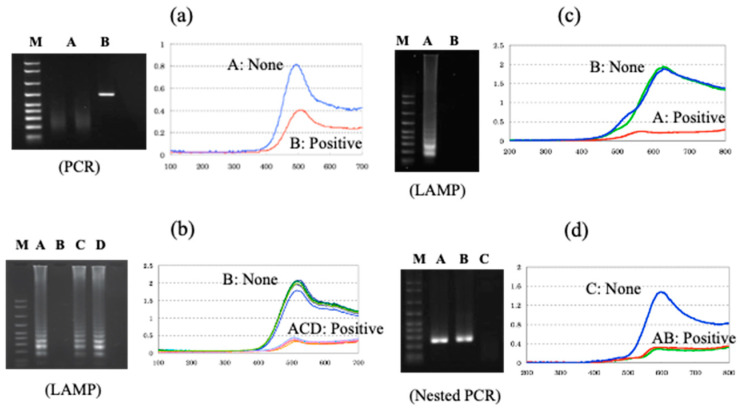
Various applications of our electrochemical DNA sensor (**a**) Salmonella gene amplified with PCR; (**b**) Pathogenic *E. coli* O157 gene amplified with LAMP; (**c**) Genetically modified maize gene amplified LAMP; (**d**) Hepatitis B virus (blood samples) gene amplified nested PCR. Each target was tested by electrochemical measurement and gel electrophoresis using positive and negative samples.

**Figure 6:**
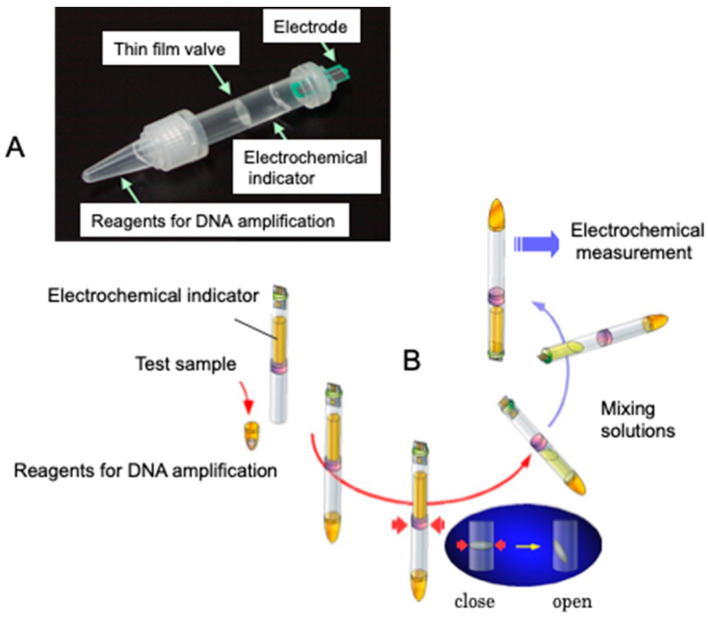
DNA stick for PCR amplification and electrochemical detection. (**A**): Structure of DNA stick; (**B**): Operation of DNA stick.

**Figure 7:**
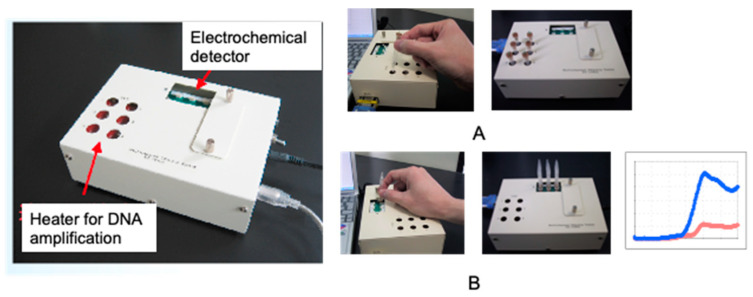
Electrochemical detector with heater for DNA amplification. (**A**): DNA stick set at heater for DNA amplification; (**B**): Electrochemical detection after operation of DNA stick shown in Figure 6.

Genetic measurements are important for the rapid diagnosis of infectious diseases, such as coronaviruses, in the field. In this context, the use of electrochemical techniques for genetic measurements has been around since 1960 [2]. Initially, the electrochemical activity of the nucleic acid molecules that make up the gene itself was the focus, and electron transfer was studied directly on the electrode. Later, many studies were carried out on the use of oligo-nucleic acid molecules placed on electrodes to form complementary bonds (hybrids), and to create sensors based on changes in the electrochemical signal. The electrochemical properties of nanomaterials, such as gold nanoparticles and graphene, were also used to amplify the signals for sensitive measurements [3,4]. Although these are mainly amperometric methods to measure electron transfer, methods have also been developed to measure gate potential changes in field-effect transistors and electrochemical impedance changes. In this way, measurements are made by placing nucleic acid molecules on electrodes. Gene amplification reactions, such as PCR, which have already been established for the detection of specific genes and DNA-binding activity with electrochemical activity, can be used to detect specific genes [5]. In Figure 2, single-strand DNA is immobilized on an electrode, and the electron transfer from the intercalating electrochemically active molecule to the electrode, when complementary DNA is bound, can be measured as an increase in the current value [6,7,8]. This has been commercialized by Toshiba Corporation [9]. Our strategy of using intercalating molecules is relatively simple because the DNA can be detected by merely mixing the intercalating molecules with the DNA in solution. That is, there is no need for immobilization on the electrode surface and/or modification of signaling molecules to nucleic acids. Detailed protocols for the intercalator-based electrochemical detection of DNA in solution have been reported [10]. The electrochemical response changes when electroactive molecules intercalate DNA through the formation of an intercalator–DNA complex, and it is dependent on the amount of amplified DNA. DNA amplification is typically performed by PCR. The choice of electrochemical indicators that can be used for this purpose is limited, because, although there are many types of DNA intercalation molecules, the electrochemical signal is very weak in most methods, and some molecules have a good electrochemical signal, but a low affinity for DNA. The most effective and widely used electroactive DNA intercalation molecules for the detection of DNA amplification are methylene blue [11,12,13], osmium–bipyridine-based complexes, bisbenzimidazole trihydrochloride (Hoechst 33258) [14,15,16], and others. The simplicity of this electrochemical method makes it suitable for the development of on-site gene detection devices. Using disposable electrochemically printed (DEP) chips, we have developed a simple intercalator-based DNA biosensor [17]. First, we screened various intercalators (about 20 different types) and observed the effect of DNA binding on their diffusion coefficients (Figure 3). Changes in diffusion coefficients have been associated with the formation of complexes between the DNA and intercalators [16]. In particular, it was observed that the binding of Hoechst 33258 to DNA significantly altered its diffusion coefficient. Furthermore, in order to confirm the presence of a specific gene, the gene was amplified by PCR and the change in the current value upon amplification was measured (Figure 4). Based on this principle, the pathogenic microorganisms involved in food poisoning, such as *Salmonella*, *E. coli*, hepatitis B virus and GMO, were examined, and the results of PCR electrophoresis were consistent (Figure 5). Biodevice Technology Ltd. has made a DNA stick with electrodes built into the reaction tube (DNA stick) in which PCR is carried out, which can be mixed with an electrochemical indicator after PCR amplification, as shown in Figure 6 [18]. They have also developed a DNA tester that integrates a temperature heater for DNA amplification and an electrochemical measuring unit as a corresponding measuring instrument (Figure 7).

## 3. Monitoring of Periodontal Disease

**Figure 8:**
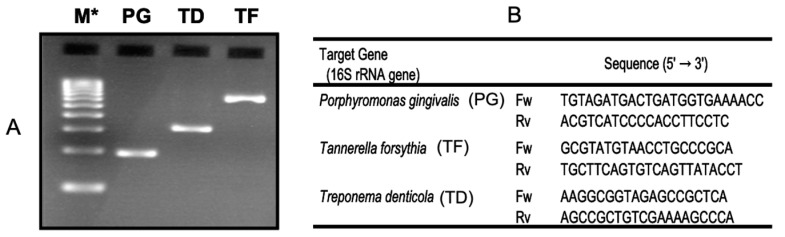
Detection of periodontal bacteria (PG, TF and TD) using PCR amplification [15] (**A**): Electrophoretic pattern of amplified PCR products. M* molecular size marker; (**B**): Sequence of PCR primers for periodontal bacteria.

**Figure 9:**
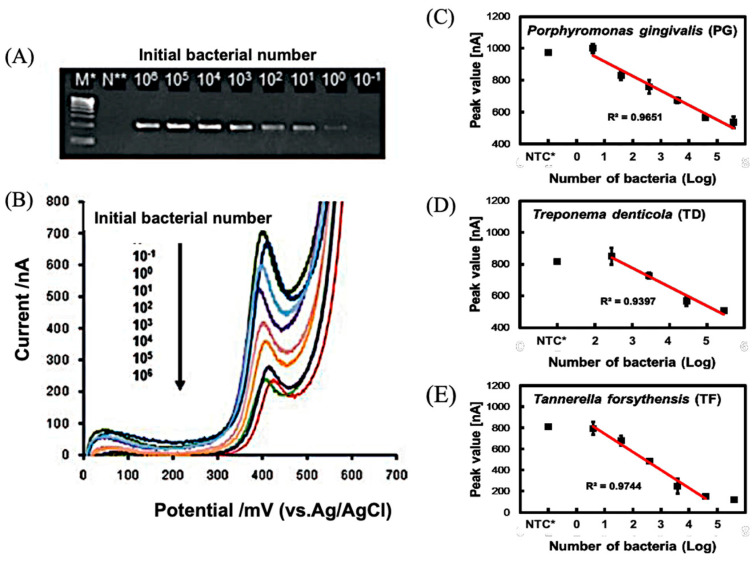
Electrochemical measurement of periodontal bacteria using PCR amplification [15]. (**A**) Electrophoresis of PCR-amplified genes encoding PG bactetium. Initial bacterial number (10^1^–10^5^), M* (molecular weight marker) and N* (negative control) were indicated. (**B**) Cyclic voltammograms(0–700 mV) were obtained with PCR amplified genes with different initial bacterial number. Calibration for number of bacteria of PG (**C**), TD (**D**) and TF (**E**).

Periodontal disease is caused by pathogenic microorganisms that live in the spaces between the teeth and gums. The major microorganisms are called red-complex bacteria, consisting of Porphyromonas gingivalis (PG), Treponema denticola (TD) and Tannerella forsythensis (TF). We investigated the use of an electrochemical DNA assay as a PCR detection method [15]. Primers were designed for the 16S rRNA gene. The sequences are shown in Figure 8, and the sizes of the targets are 197 bp (PG), 311 bp (TD) and 641 bp (TF), which are presented by electrophoretic patterns (Figure 8). We performed direct PCR using diluted cultures, in order to prepare a calibration curve for the number of PG bacteria. The target sequence of the PCR was 16S rDNA, which is specific for PG. The increase in the PCR product with the number of bacteria was confirmed by electrophoresis, and corresponded to the decrease in the current peak in the electrochemical measurement (Figure 9A,B). The calibration curve compares the real-time PCR with the electrochemical method, as shown in Figure 9C–E. In the electrochemical method, the measurement was performed after 40 cycles (endpoint assay). The electrochemical calibration curve became saturated when the number of bacteria increased, but this was due to the high number of cycles, and it was presumed that the calibration characteristics could be further controlled by controlling the number of cycles. In the case of high concentrations, it was also possible to cover the measuring range by dilution.

**Figure 10:**
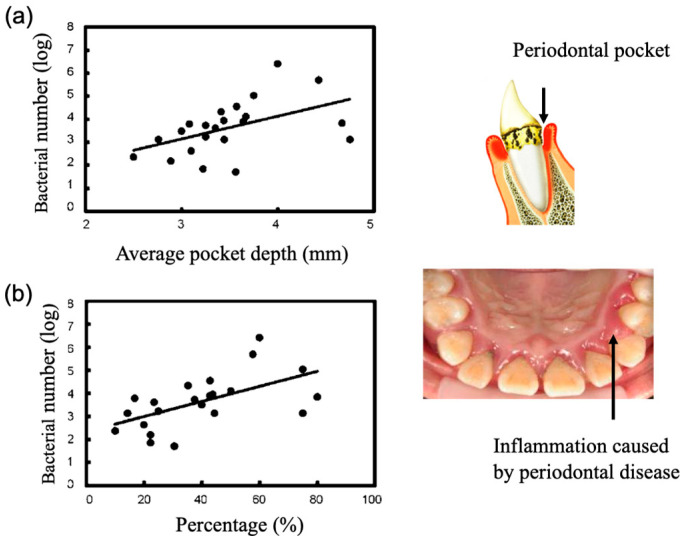
Relationship between periodontal pocket depth and PG bacterial number estimated by our electrochemical method [15]. (**a**) Plot between the number of PG bacteria and the average pocket depth; (**b**) Plot between the number of PG bacteria and the percentage of the periodontal teeth in which the periodontal pocket depth was greater than 4 mm.

**Figure 11:**
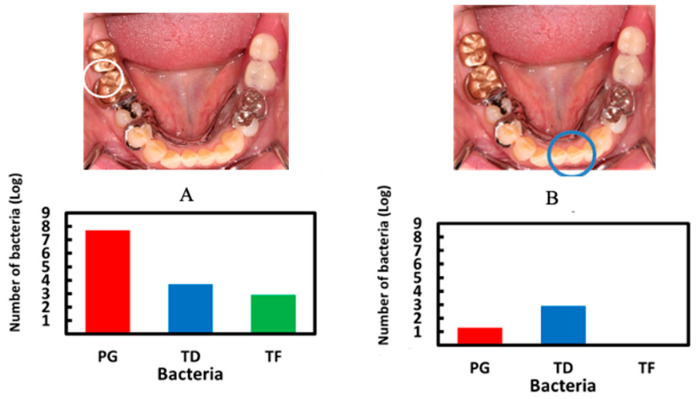
Bacterial number profiles between deepest (**A**) and shallowest (**B**) pockets. White and blue circles indicated deepest and shallowest pockets, respectively. PG, TD and TF were periodontal bacteria.

**Figure 12:**
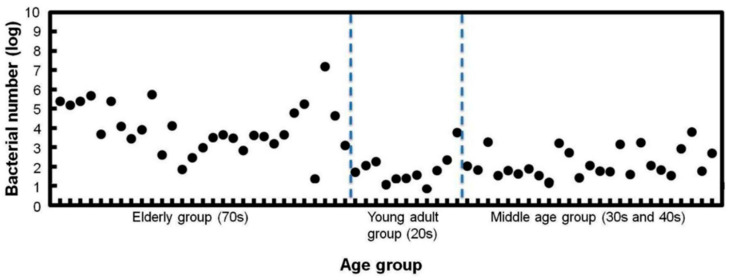
Electrochemical measurement of periodontal bacteria in saliva with different age groups [15]. Saliva samples were collected from 20s group (18 people) 30~40s group (24 people) 70s~group (33 people).

Next, we investigated the use of saliva. Saliva sampling is a simple and non-invasive sampling method that is not affected by skill level. Therefore, in order to develop a more convenient periodontal disease test method, we performed quantitative PG detection by directly collecting saliva, and we examined the relationship between the number of PG bacteria and the average periodontal pocket depth of each subject. The number of PG bacteria in the saliva was related to the average depth of the periodontal pockets (Figure 10a). In general, periodontal disease is clinically diagnosed in patients with a pocket depth of 4 mm or more. Therefore, we investigated the relationship between the number of PG in saliva and the percentage of teeth with periodontal pocket depths of 4 mm or more. The number of PG in the saliva showed a significant correlation coefficient of 0.88 with the percentage (Figure 10b). Next, periodontal fluid samples from different depths of periodontal pockets were taken to compare the type and amount of bacteria. Healthy teeth with a depth of 2 mm and covered teeth with a depth of 9 mm were compared. The results showed that all three bacteria were present in high concentrations in the periodontal fluid of the teeth with deeper pockets. PG bacteria were particularly abundant. On the other hand, in the periodontal fluid with shallow pockets, the number of PG bacteria was considerably reduced, and the number of TF bacteria was reduced to below the measurement limit (Figure 11). On the other hand, the proportion of infected individuals diagnosed with periodontal disease is known to be strongly dependent on age, and periodontal disease has been clinically classified as an age-dependent disease. Therefore, we compared the results of the number of PG bacteria in saliva of different age groups. As shown in Figure 12, the mean number of PG in the saliva of the middle-aged group (30 s and 40 s) was higher than that of the younger group (20 s). The average number of PG in the elderly group (in their seventies) was much higher than in the other two groups. Thus, there was a strong dependence between the number of PG and the age group, which was consistent with clinical observations. These results indicate that the quantitative detection of PCR-amplified products of PG in saliva clearly reflects the degree of periodontal disease and its age dependence, and that our electrochemical detection method can be used as a simple screening method for PG bacteria for the diagnosis of periodontal disease. It should provide a concise and precise description of the experimental results, their interpretation, as well as the experimental conclusions that can be drawn.

## 4. Monitoring of MRSA Bacteria

**Figure 13:**
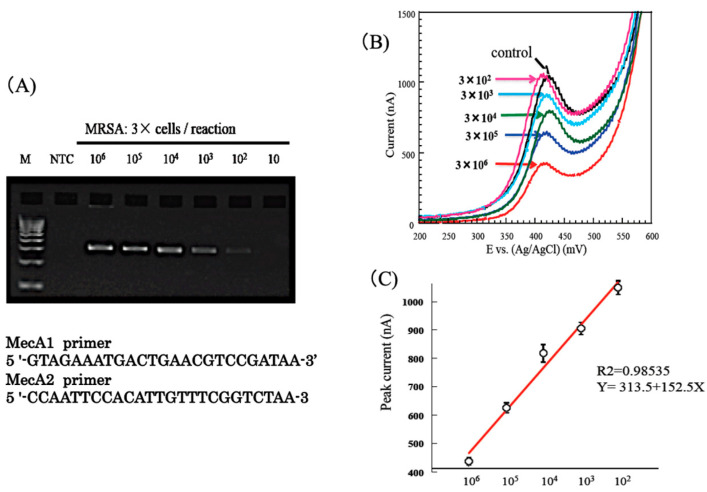
Monitoring of nosocomial MRSA bacteria [19]. (**A**) Electrophoresis of PCR-amplified genes encoding MRSA; (**B**) Cyclic voltammograms (0–600 mV) were obtained with PCR amplified genes with different initial bacterial number; (**C**) Calibration of bacterial number with peak current.

**Figure 14:**
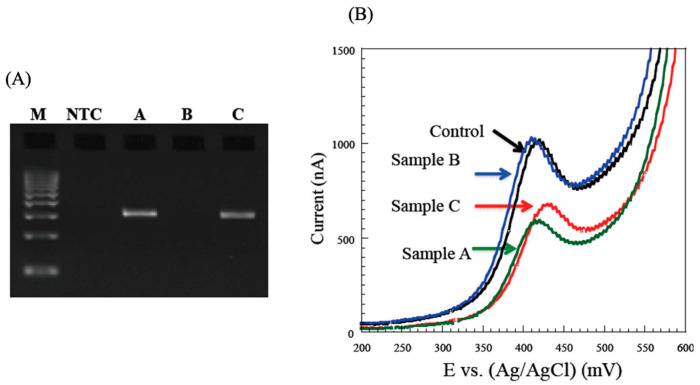
Detection of MRSA from patient samples [19]. (**A**) Electrophoresis of PCR-amplified genes encoding MRSA. Patient samples of A,C (positive) and B (negative) NTC: negative control; (**B**) Cyclic voltammograms (0–600 mV) were obtained with PCR amplified genes with patient samples.

*Staphylococcus aureus* is found on human skin, nasal cavities, and other body surfaces, and is usually harmless to healthy people, but infection can occur in the presence of wounds. *Staphylococcus aureus*, which is resistant to the antibiotic methicillin (MRSA), can cause respiratory tract infections, bacteremia, and postoperative infections in immunocompromised patients, through transmission in hospitals. The resistance function of MRSA is due to the inducible production of penicillin-binding protein. The structural gene that controls the production of this protein is *mec*A. The presence of the *mec*A gene in *Staphylococcus aureus* can be used to determine whether the organism is MRSA or not. We performed PCR amplification of the *mec*A gene using MRSA isolates, and, after 40 cycles of PCR, the products were subjected to agarose gel electrophoresis (Figure 13A) [19]. Specific amplified product (310 bp) was clearly observed in four solutions containing 3 × 10^6^ to 3 × 10^3^ MRSA. When this sample was subjected to electrochemical measurements, the current decreased with increasing bacterial concentration (Figure 13B) [19]. A calibration curve could be constructed from the relationship between the peak current value and the number of MRSA bacteria (Figure 13C) [19]. The regression coefficient was 0.985. This result suggests that the MRSA bacterial count can be directly quantified with high sensitivity and accuracy, without DNA extraction. The time required for the electrochemical measurement after amplification was less than 2 min. A potential clinical application of the method was then investigated using nasal swabs collected from patients. The nasal swabs were subjected to PCR, followed by electrochemical measurements, to determine the amount of colony-forming MRSA. Nasal swabs A, B, C and NTC were subjected to PCR and subsequent gel electrophoresis (Figure 14A) [19]. Specific amplified product (310 bp) was clearly visible in samples A and C, but not in NTC and sample B. The results of electrochemical current measurements of the same PCR mixtures are shown in Figure 14B [19]. These results were in agreement with the semi-quantitative results obtained by culturing the samples in a hospital laboratory (Supplementary Materials Table S1).

## 5. Rapid Influenza Virus Gene Sensor Using a Chip with Microfluidic PCR

**Figure 15:**
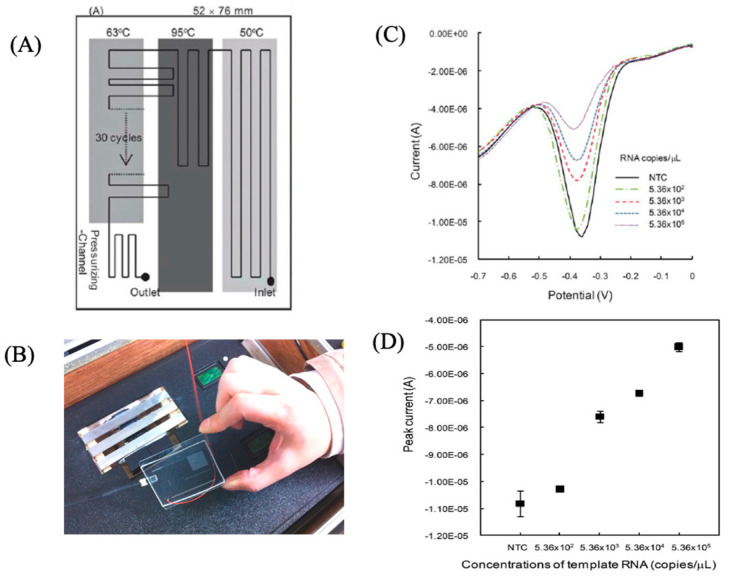
RT-PCR microfluid chip with screen printed electrode [12]. (**A**) Microfluidic RT-PCR chip with fixed temperature heaters of 63 °C, 95 °C and 50 °C; (**B**) Photograph of (**A**); (**C**) Square Wave Voltammetry of RT-PCR products of different copies of RNA samples; (**D**) Relationship between of peak currents and RT-PCR products of different copies of RNA samples.

Subtyping by reverse transcription–polymerase chain reaction (RT-PCR) has been established as a routine diagnostic method for RNA viruses, such as new coronaviruses and influenza. We fabricated a microfluidic chip for rapid RT-PCR, and investigated a method for the electrochemical measurement of amplified products using printed electrodes. To measure the electrochemical signal, methylene blue (MB) was added to the RT-PCR mixture and measured by SWV (square-wave voltammetry) [12]. Electrochemically active molecules were used to detect the DNA amplified by PCR. Hoechst bound to the small groove in the AT-rich region of the DNA. It is known that MB has a high affinity for nucleic acids, and that the affinity differs between single-stranded DNA and double-stranded DNA. The RT-PCR chip we fabricated consisted of the following four zones: an RT reaction zone (50 °C), DNA thermal denaturation zone (95 °C), DNA elongation reaction zone (2-step PCR), and a pressurized flow zone to prevent bubble generation. The entire distribution time, from the inlet to the outlet, was completed within 15 min, and electrochemical measurements could be performed quickly. The microfluidic chip for fast RT-PCR and the printed electrode chip for electrochemical sensing are suitable for integration, and can be used as a portable genetic measurement system that can be performed quickly in the field (Figure 15).

## 6. Electrochemical Gene Sensor Using Isothermal Gene Amplification Reaction

**Figure 16:**
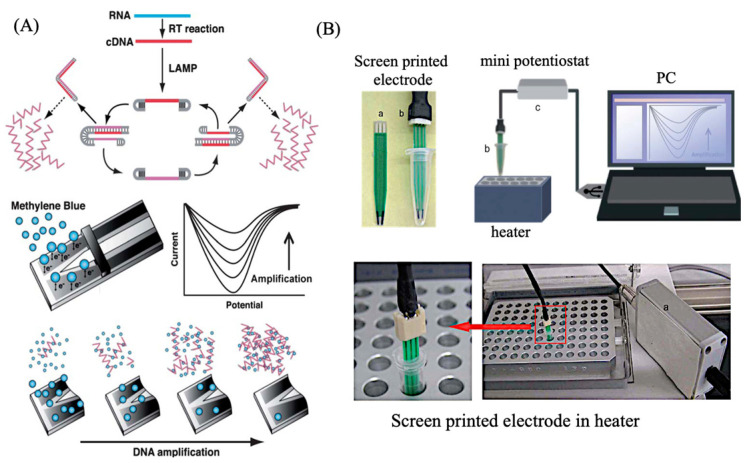
Real-time electrochemical measurements using isothermal gene amplification [20]. (**A**) Scheme of electrochemical detection of LAMP amplified DNA; (**B**) Electrochemical measurement system with screen printed electrodes and heater.

**Figure 17:**
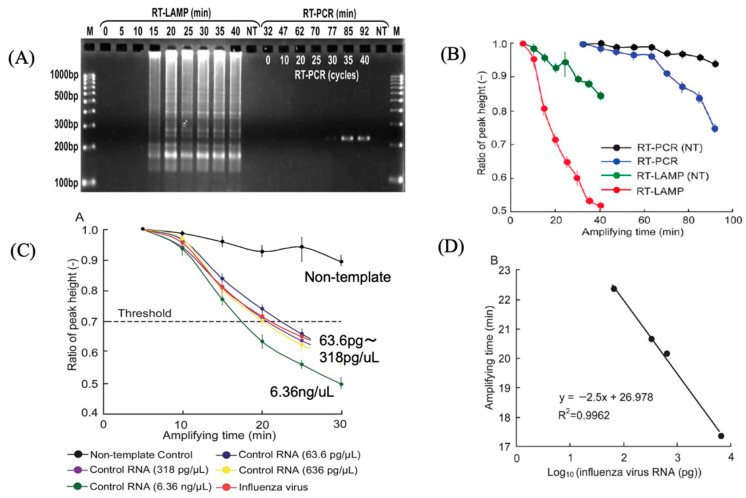
Influenza virus measurement using isothermal gene amplification [20]. (**A**) Electrophoresis of RT-LAMP and RT-PCR-amplified genes encoding Influenza virus (**B**) Comparison of electrochemical responses of RT-LAMP and RT-PCR (**C**) Electrochemical responses with different copies of RNA and influenza sample (**D**) Calibration of influenza RNA.

This gene sensor is based on electrochemical monitoring of the amplification of specific genes, and can be linked to various types of gene amplification reactions; PCR is the best-known method of gene amplification, but it requires temperature cycling control, special heaters, and the microfluidic chips mentioned above. On the other hand, the isothermal amplification method described here requires a short amount of time at a certain temperature, which is very advantageous for the development of POCT diagnostic devices. An example of the development of a gene sensor was shown using the LAMP method. The LAMP reaction is an automated cyclic strand-displacement reaction using a highly active Bst DNA polymerase that can rapidly amplify several copies of DNA in a single reaction tube. Unlike PCR, the LAMP reaction requires four primers that are designed to recognize six different sequences, which allows the amplification of target sequences with high selectivity. Figure 16A shows the principle of electrochemical monitoring of DNA amplification using MB [20]. Upon binding of the MB molecule to the amplified DNA, the MB-amplified DNA complex slowly diffuses to the electrode surface, reducing the peak current. By connecting the screen-printed electrode to a USB-powered portable potentiostat, and measuring and analyzing the electrochemical signal of MB, we monitored the amplification process of DNA based on RT-LAMP (Figure 16B) [20]. A comparison of the time required for amplification between the RT-LAMP and RT-PCR methods shows that the RT-LAMP method takes 15 min and the RT-PCR method takes 85 min, with a higher amplification rate for the LAMP method (Figure 17A) [20]. Here, the amplification of DNA by the reverse transcription (RT)–LAMP method, using MB, was monitored sequentially. The results showed that the electrochemical response changed with the amplification time and RT-LAMP could amplify more rapidly (Figure 17B) [20]. In addition, referring to the method of quantification, by setting the threshold value to that used in real-time PCR, when the threshold value was set at 0.7, the ratios of 63.6 pg/mL, 318 pg /mL, 636 pg/mL, and 6.36 ng /mL were 22.4 min, 20.7 min, 20.2 min, and 17.4 min, respectively, from the threshold value (Figure 17C) [20]. The calibration of virus RNA is also shown in Figure 17D [20].

The immunochromatographic method is currently used in medical practice for the diagnosis of influenza, but it has been pointed out that it has low sensitivity and is difficult to diagnose, especially in the early stages of infection. In this study, we compared the sensitivity of the immunochromatographic method with that of this method, using leftover specimens from patients after immunochromatographic diagnosis, in cooperation with a clinic. Five specimens that had tested negative with immunochromatography were found to be positive, demonstrating the superior sensitivity of the immunochromatography method. In addition, the use of a genetic diagnosis method enabled a more accurate diagnosis, in terms of virus typing (Supplementary Materials Table S2).

In addition to LAMP, other methods, such as RPA (recombinase polymerase reaction), NEAR (nicking enzyme amplification reaction) and RCA (rolling circle amplification), are also known for isothermal amplification, some of which amplify at room temperature and body temperature, making it possible to design devices that do not require a heater. There are also chemical heaters that use the oxidative energy of magnesium or iron, which can be used in environments where there is no electrical power source [21].

## 7. SNPs Sensor

SNPs are genetic changes or variations that may occur in an individual’s DNA sequence, and these variations between individuals are thought to influence disease susceptibility and treatment efficacy. Such inter-individual variation is thought to determine susceptibility to disease and responsiveness to treatment. SNPs in ADH activity, which is associated with alcohol sensitivity, and in the drug-metabolizing enzyme cytochrome P450, which is associated with drug sensitivity, are examples. Apolipoprotein E (ApoE), one of the genes associated with Alzheimer’s disease, has two SNPs, resulting in three alleles, E2, E3 and E4. Each allele differs by one base and one amino acid in the protein product of each gene. SNPs are not absolute indicators of disease development, but the relative risk is known (Supplementary Materials Table S3) [22]. This was applied to the diagnosis of ApoE types. Primers that are specific for this allele were used, PCR was performed, and electrochemical measurements were made. Anonymous DNA samples from consenting adults were used. Amplified C/T positive and C/T negative were measured on a DEP chip. If a human carried a gene with positive T and C nucleotides at the codon position Cys112Arg, and only the T nucleotide was negative and the C nucleotide was positive at the codon position Arg158Cys, the person was determined to be the E3/E4 genotype. Similarly, if all the nucleotides at that particular codon position were positive, the person would be the E2/E4 genotype (Supplementary Materials Table S4) [22]. In this way, it was possible to easily diagnose the risk factors for Alzheimer’s disease. In other words, it was possible to diagnose SNPs based on the pattern of gene amplification using PCR primers corresponding to the SNPs in easily collected biological samples, such as oral mucosa or saliva. Here, we have been able to identify six different SNP patterns using four different gene sensors.

## 8. Conclusions

The viewpoint of this paper is that our method is advantageous for monitoring DNA amplification reactions in solution, without modifying DNA probes on the electrodes, as shown in Figure 2. In addition, an electrochemical measurement system with printed electrodes and portable measurements has been developed, which is an advantage of this method for POCT diagnosis. As for the gene amplification method, a microfluidic device that allows multiple rapid temperature cycles of PCR has also been developed (Figure 15). In addition, the use of isothermal amplification, such as LAMP, simplifies the equipment and makes POCT diagnostic devices more feasible. We suggest that these portable genetic diagnostic devices will be useful as routine diagnostic devices and as health care devices in the home. It would be extremely useful to be able to easily diagnose infectious conditions, such as periodontal disease and influenza virus, using saliva and nasal fluid, as shown in this paper.

## Data Availability

Data is contained within the article or Supplementary Material.

## Supplementary Materials

The following supporting information can be downloaded at: https://www.mdpi.com/article/10.3390/s22051865/s1. Figure S1. Features and advantages of screen printed electrodes. Table S1. Comparison between electrochemical and culture results regarding estimate of MRSA number in clinical samples [19]. Table S2. Electrochemical RT-LAMP tests with patient samples. Table S3. Apolipoprotein gene (ApoE) polymorphism and risk of Alzheimer disease [22]. Table S4. Electrochemical results with real human samples [22].
