# Supplementary material for: Portable Electrochemical DNA Sensors Based on Gene Amplification Reactions to Screen and Identify Pathogen and SNPs
Source: Sensors (Basel). 2022 Feb 26;22(5):1865. doi: 10.3390/s22051865 (PMC8914808; doi:10.3390/s22051865)
Supplement: Supplementary file 1 [file sensors-22-01865-s001.zip › sensors-1579755-supplementary.pdf]

Supplementary Materials Figure S1

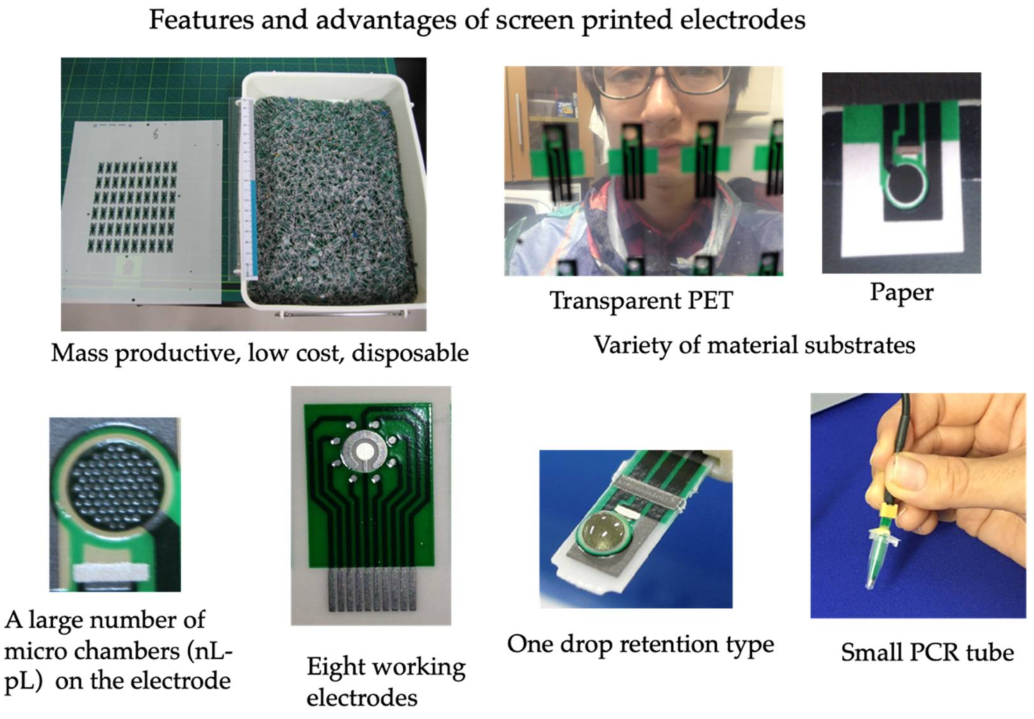

Supplementary Materials Table S1

Comparison between electrochemical and culture results regarding estimate of MRSA number in clinical samples [19]

Sample	Estimated MRSA number(cells/mL)	Culture results
A	3.8×10^5	++
B	Negative	-
C	1.0×10^5	+
+: < 100 colonies /plate, ++: 100-500 colonies/plate, +++: 500> colonies /plate		

Supplementary Materials Table S2

Electrochemical RT-LAMP tests with patient samples

Patient sample	Immunochromatography	RT-LAMP
T1	(B+)	(B+)
T2	(—)	(—)
T3	(—)	(—)
T6	(B+)	(—)
T14	(—)	(—)
T15	(A+)	A/H3N2+
T16	(—)	(—)
T17	(—)	(—)
T18	(—)	(—)
T20	(—)	(—)
T22	(—)	(—)
M1	(—)	(—)
M8	(—)	(—)
M10	(—)	(—)
M11	(—)	(—)
M12	(—)	(—)
M21	(—)	(—)
M26	(—)	(—)
M40	(—)	(—)
M42	(—)	(—)
M43	(—)	(—)
M45	(—)	(—)
M46	(—)	(—)
M47	(—)	(—)
M51	(—)	(—)
M52	(—)	A/H3N2+(RT-PCRでもA+)
M53	(—)	(—)
M54	(—)	(—)
M55	(—)	(—)
M57	(—)	A/H3N2+
M64	(—)	(—)
M65	(—)	(—)
M66	(—)	A/H3N2+
M68	(—)	(—)
M69	(—)	A/H3N2+
M70	(—)	(—)
M71	(A+)	A/H3N2+
M72	(—)	(—)
U1	(A+)	A/H3N2+
U2	(A+,B+)	A/H3N2+, B+
U3	(—)	(—)
U4	(—)	A/H3N2+
U6	(—)	(—)
U8	(—)	(—)

Yellow boxes indicated negative by immunochromatography and positive by electrochemical RT-LAMP

Supplementary Materials Table S3

Apolipoprotein gene (ApoE) polymorphism and risk of Alzheimer disease [22]

Genotypes	SNP positions: 334 (Cys112Arg)	SNP positions: 472 (Arg158Cys)	Nucleotides 334: T	Nucleotides 334: C	Nucleotides 472: T	Nucleotides 472: C	(Relative Risk of Onset)
E 2/2	—T—	—T—	+	−	+	−	(0.6)
	—T—	—T—					
E 2/3	—T—	—T—	+	−	+	−	
	—T—	—C—					
E 2/4	—T—	—T—	+	+	+	+	(3.2)
	—C—	—C—					
E 3/3	—T—	—C—	+	−	−	+	(1.0)
	—T—	—C—					
E 3/4	—T—	—C—	+	+	−	+	(3.2)
	—C—	—C—					
E 4/4	—C—	—C—	−	+	−	+	(11.6)
	—C—	—C—					

Supplementary Materials Table S4

Electrochemical results with real human samples [22]

	Cys112Arg (T/T) Anodic peak/nA (SD) B–C		Cys112Arg (T/T) DNA detected[a]	Cys112Arg (C/C) Anodic peak/nA (SD) A–C		Cys112Arg (C/C) DNA detected[a]	Arg158Cys (T/T) Anodic peak/nA (SD) E–F		Arg158Cys (T/T) DNA detected[a]	Arg158Cys (C/C) Anodic peak/nA (SD) D–F		Arg158Cys (C/C) DNA detected[a]	ApoE genotype
1	78.33	2.31	+	95.64	1.55	−	106.75	6.89	−	73.32	2.47	+	E3/E3
2	73.78	2.06	+	73.29	2.43	+	102	3.605	−	80.36	1.71	+	E3/E4
3	79.6	1.59	+	69.95	2.94	+	98.33	1.29	−	77.22	2.43	+	E3/E4
4	76.67	1.155	+	79.76	1.763	+	103.56	2.157	−	68.67	2.58	+	E3/E4
5	73.77	3.276	+	69.62	2.44	+	109.4	1.357	−	58.5	1.87	+	E3/E4
6	72.45	2.52	+	78.99	1.3	+	101.5	2.871	−	77.5	0.619	+	E3/E3
7	73.47	2.64	+	81.31	0.814	+	80.315	1.96	+	75.87	3.85	+	E2/E4
8	80.98	0.541	+	98.45	2.61	−	102.93	3.42	−	65.86	1.143	+	E3/E3
9	77.24	3.19	+	75.11	0.88	+	106.26	1.686	−	72.26	3.159	+	E3/E4
10	75.51	1.52	+	76.02	1.82	+	68.28	1.91	+	70.43	6.1	+	E2/E4
